# Numerical Simulation Study on the Impact of Blind Zones in Ground Penetrating Radar

**DOI:** 10.3390/s25041252

**Published:** 2025-02-18

**Authors:** Wentian Wang, Wei Du, Siyuan Cheng, Jia Zhuo

**Affiliations:** 1 Institute of Disaster Prevention, Sanhe 065201, China; wwt@cidp.edu.cn; 2 Hebei Key Laboratory of Earthquake Dynamics, Sanhe 065201, China; 3 School of Earth Science, Yunnan University, Chenggong Campus, Kunming 650091, China; 4 WSGRI Engineering & Surveying Incorporation Limited, Wuhan 430000, China; chengsy6@mail2.sysu.edu.cn; 5 School of Civil Engineering, Changsha University of Science & Technology, Changsha 410114, China

**Keywords:** GPR, borehole radar, blind zones, FDTD, numerical simulation

## Abstract

Ground-penetrating radar (GPR) is an effective geophysical method for rapid and non-destructive detection. Directional borehole radar is the application of GPR in a borehole, which can determine the depth, orientation, and distance of the target from the borehole. The borehole radar azimuth recognition algorithm is based on the assumption of far-field plane waves. Therefore, in the near-field area where the target is closer to the borehole, the electromagnetic waves reflected by the target cannot be regarded as plane waves but will have a certain curvature. The plane wave assumption is not valid in this area, so the azimuth recognition algorithm will have significant errors, forming blind zones for directional borehole radar detection. This article uses the finite-difference time-domain (FDTD) algorithm to numerically simulate how blind zones affect directional borehole radar systems, identify the impact patterns, and minimize them. After calculation and numerical simulation verification, it has been found that when the center frequency of the antenna is 1 GHz, within 2 m of the target from the borehole, there is a significant error in azimuth recognition, which can be defined as the near-field region. Similarly, through numerical simulation verification, the optimal antenna center frequency is between 600 MHz and 1100 MHz. Oil-based mud is superior to water-based mud. The optimal antenna center frequency decreases as the target distance increases.

## 1. Introduction

Ground-penetrating radar (GPR) is a non-destructive exploration method that utilizes high-frequency ultra-wideband signals to detect the distribution patterns of shallow subsurface media. The working principle of GPR technology is as follows: First, ultra-wideband electromagnetic waves are transmitted underground. When the electromagnetic waves encounter heterogeneous bodies with different dielectric properties, electromagnetic phenomena such as reflection, refraction, and scattering occur at the interface between adjacent media, and the reflected signals are received by the GPR receiving antenna. Since 1930, GPR technology has developed rapidly, achieving significant progress in both theoretical and methodological aspects, as well as in various related fields of application [1]. Due to its characteristics of convenient construction, high detection efficiency, and high resolution of detection results, the application of GPR technology has shown a diversified trend and has been widely used in civil and military fields such as archaeology [2,3,4], environmental engineering [5], geological issues [6,7], municipal engineering [8,9], earth science [10,11], and landmine detection [12]. However, since ground-penetrating radar employs high-frequency electromagnetic waves for detection, which have the drawback of shallow detection depth, borehole radar has been developed.

Borehole radar is the application of ground-penetrating radar in a borehole, allowing it to get closer to the target and address the issue of insufficient detection depth in surface radar [12,13]. However, borehole radar can only determine the depth of the target and its distance from the borehole but cannot pinpoint the specific location of the target [14,15]. To solve this problem, a series of boreholes would be needed, which would incur significant additional economic costs. Therefore, to address this issue, directional borehole radar has been developed.

**Figure 1:**
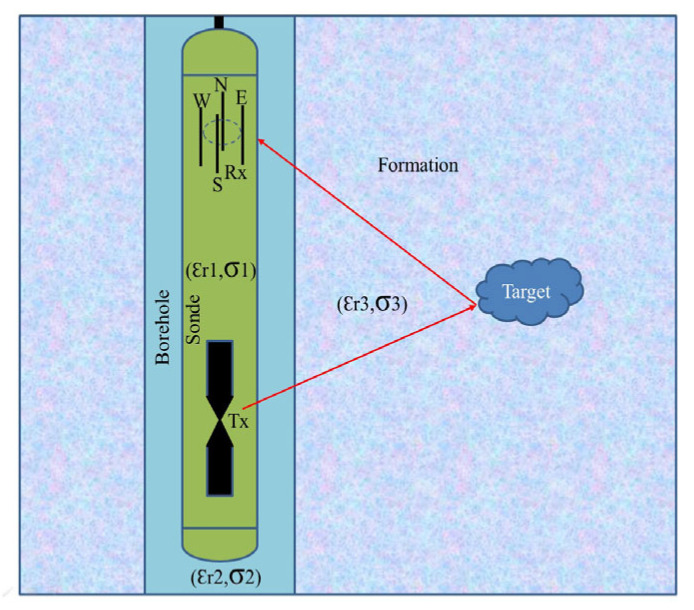
Directional borehole sonde with one transmitting and four receiving antennas forming a uniform circular array (UCA), located in a water-filled borehole.

Directional borehole radar can determine the target’s location within a single hole, significantly reducing economic costs [16,17,18]. There are two types of directional borehole radar: directional transmitting systems and directional receiving systems. The directional borehole radar discussed in this article is a directional receiving system. Directional receiving antennas often use array antennas, which consist of one transmitting antenna and four receiving antennas. The four receiving antennas are located in the south, north, east, and west directions, respectively, as shown in Figure 1. The slight differences in the waveforms received by the four receiving antennas are used for location identification [19,20]. However, previous azimuth identification algorithms were all based on the far-field assumption. Therefore, the target needs to be sufficiently far from the borehole, and the waves reflected by the target can be regarded as plane waves. That is to say, the far field here refers to the array radius of the directional borehole radar receiving antenna, rather than the wavelength in the traditional sense of electromagnetic waves. However, for directional borehole radar systems, many targets are located in the near-field region close to the borehole. Therefore, it is necessary to clarify the impact of blind zones on azimuth identification through numerical simulation, find patterns, and minimize this impact.

Within the near-field range, the non-uniformity around the borehole and the antenna system of the directional borehole radar can affect signal reception. I have previously discussed in my paper the effects of the borehole, the relative permittivity of the fluid inside the borehole, and the array diameter of the receiving antenna on the signal reception of the directional borehole radar [21,22]. Simulation results show that when the relative permittivity of the fluid inside the borehole is high, strong resonance occurs. Although the resonance can barely allow for azimuth identification, it makes it difficult to determine the distance from the target to the borehole. However, my previous work was all based on targets in the far-field region, and there are significantly different patterns in the blind zones of the near-field. At the same time, my previous work did not explore the effects of the frequency of the receiving antenna and the distance from the target to the borehole on the receiving antenna. Through this study, it is known that when the distance from the target to the borehole varies, especially when the target is in the near-field blind zone, the most suitable antenna frequency is significantly different from that in the far-field.

Therefore, this numerical simulation study focuses on the near-field blind zone of directional borehole radar, aiming to identify the patterns of how various factors within this zone affect the received signals of the radar and to propose solutions to minimize the adverse effects.

Through numerical simulation research in this article, we can draw the following conclusions. Selecting the north direction as 0° and the clockwise direction as the positive direction, the azimuth recognition error is zero when the target azimuth angle is 45° or a multiple of 45°. The maximum azimuth recognition error occurs when the target azimuth angle is 22.5° or an odd multiple of 22.5°. The center frequency of the antenna will significantly affect the received signal of directional borehole radar, and a low antenna frequency will result in low signal strength. Excessive antenna frequency can cause multiple waves between the borehole and the target to be more pronounced, resulting in significant resonance. Through numerical simulation verification, the optimal frequency of the antenna is between 600 MHz and 1100 MHz. Finally, the fluid in the borehole will also have a significant impact on the received signal. Numerical simulations have verified that when the relative permittivity of the fluid in the borehole is higher than 15, the received signal will resonate significantly. Therefore, using oil-based mud will be significantly better than water-based mud. When the distance between the target and the borehole is different, the most suitable antenna center frequency is also different. The farther the target is, the lower the most suitable antenna center frequency.

## 2. Materials and Methods

### 2.1. Materials

The borehole model for this numerical simulation takes into account the borehole, probe tube, and borehole fluid. The borehole fluid is either oil-based mud or water-based mud. Depending on the ratio of oil to water or mud to water, the relative permittivity varies between 3 and 80. The probe tube is a PVC tube with a relative permittivity of 4 and a conductivity of 0.0001. The surrounding rock is granite, with a relative permittivity of 5 and a conductivity of 0.0007. The target filler is water, with a relative permittivity of 80 and a conductivity of 0.7. Both the transmitting antenna and the receiving antenna of the directional borehole radar system are point antennas.

### 2.2. Methods

#### 2.2.1. Finite-Difference Time-Domain Algorithm for Electromagnetic Waves

Solving Maxwell’s equations is an effective method for addressing electromagnetic problems. However, Maxwell’s equations involve a large number of derivative operations, making it relatively difficult for computers to directly calculate the derivatives. The essence of derivation is to find the slope of the tangent at a given point. Therefore, we use the slope of the secant line to replace the slope of the tangent. When the distance between two points is relatively close, the slope of the secant line can be approximated as the slope of the tangent. This is the idea of finite difference. The finite-difference time-domain (FDTD) algorithm for electromagnetic waves uses this difference idea to solve Maxwell’s equations [22,23]. As a major time-domain computational method for electromagnetic fields, FDTD was first proposed by K.S. Yee in 1966 [24]. The main idea of the FDTD method is to divide the research area (i.e., the real area, including the target object and free space) into a certain number of spatial grids, approximate Maxwell’s equations using finite difference methods, then perform time discretization, and finally add corresponding initial and boundary conditions. Then, the problem can be solved by advancing in time steps.

#### 2.2.2. Basic Principles of the MUSIC Algorithm

The multiple signal classification (MUSIC) algorithm is an effective method for azimuth identification in directional borehole radar [16,17]. The azimuth identification algorithm used in this paper is the MUSIC algorithm. The MUSIC algorithm utilizes the orthogonality between the signal subspace and the noise subspace to construct a spatial spectrum function. Through spectral peak search, it detects the direction of arrival (DOA) of the signal, with the peak corresponding to the target azimuth [25,26,27,28,29]. The MUSIC algorithm is based on the following assumptions: (1) the signal source is located in the far field, and the signals emitted or reflected by the signal source and reaching the receiver can be regarded as plane waves or parallel waves; (2) the spacing between array elements is not less than half the wavelength of the highest frequency signal being processed; (3) the noise in the processor follows an additive Gaussian distribution; (4) the number of signal sources is less than the number of array elements, and the number of signal samples is greater than the number of array elements; (5) the signal sources must be uniformly distributed random numbers.

**Figure 2:**
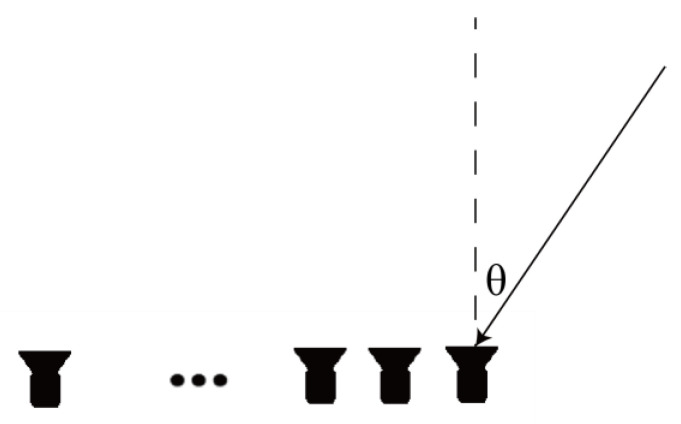
Schematic diagram of a uniform linear array.

The MUSIC algorithm is based on uniform lines, and the schematic diagram of a uniform linear array is shown in Figure 2. The mathematical model of the direction of arrival for narrowband far-field signals is as follows:

(1) $$X\left(t\right)=A(\theta )S(t)+N(t)$$

where *X(t)* is the matrix composed of signals received by the receiving antenna, *A(θ)* is the array steering vector, *S(t)* is the waveform of the signal, and *N(t)* is the noise term. The covariance matrix of array data is as follows:

(2) $$\begin{array}{cc} R & =E\left[XX^{H}\right]\\ & =AE\left[SS^{H}\right]A^{H}+\sigma^{2}I\\ & =AR_{S}A^{H}+\sigma^{2}I \end{array}$$

Here, $E\left[\cdot \right]$ denotes the calculation of expectation, ${\left[\cdot \right]}^{H}$ represents the calculation of conjugate transpose, *I* stands for the identity matrix, and *σ* indicates the variance corresponding to noise. Since the signal and noise are independent of each other, the data covariance matrix can be decomposed into two parts related to the signal and noise, where $R_{S}$ is the covariance matrix of the signal and $AR_{S}A$ is the signal part.

The eigen decomposition of *R* is as follows:

(3) $$R=U_{S}\sum_{S}U_{S}^{H}+U_{N}\sum_{N}U_{N}^{H}$$

In the formula, $U_{S}$ represents the subspace spanned by the eigenvectors corresponding to the large eigenvalues, which is also known as the signal subspace. Conversely, $U_{N}$ denotes the subspace spanned by the eigenvectors corresponding to the small eigenvalues, representing the noise subspace. $\sum_{S}$ and $\sum_{N}$ are the diagonal matrices corresponding to the signal subspace and noise subspace, respectively. Under ideal conditions, the signal subspace and noise subspace in the data space are orthogonal to each other, meaning that the steering vectors within the signal subspace are also orthogonal to the noise subspace.

(4) $$a^{H}\left(\theta \right)U_{N}=0$$

The classic MUSIC algorithm is precisely proposed based on the aforementioned property. However, considering that the actual received data matrix is of finite length, the maximum likelihood estimate of the data covariance matrix is as follows:

(5) $$\hat{R}=\frac{1}{L}\sum_{i=1}^{L}XX^{H}$$

Performing eigendecomposition on the above formula can calculate the noise subspace eigenvector matrix. Due to the presence of noise, the steering vectors in the signal subspace are not completely orthogonal to the noise subspace, which means that Formula (4) does not hold. Therefore, in practice, DOA estimation is achieved through minimum optimization search:

(6) $$\theta_{MUSIC}=arg_{\theta }min\;a^{H}\left(\theta \right){\hat{U}}_{N}{\hat{U}}_{N}^{H}\;a(\theta )$$

Therefore, the spectral estimation formula of the MUSIC algorithm is as follows:

(7) $$P_{MUSIC}=\frac{1}{a^{H}\left(\theta \right){\hat{U}}_{N}{\hat{U}}_{N}^{H}\;a(\theta )}$$

Therefore, the azimuth angle *θ* corresponding to the maximum value calculated by Formula (7) is the target azimuth.

## 3. Results

This article employs computational and numerical simulation methods to ascertain the influence patterns of the near field on received signals. Initially, the scope of the near field and the influence patterns of azimuth on received signal errors are determined through calculations. Subsequently, numerical simulations are conducted using the FDTD algorithm to ascertain the influence patterns of antenna center frequency, relative permittivity of the fluid inside the well, distance, and the distance between the transmitter and receiver on received signals. Finally, conclusions are drawn to identify a solution that minimizes adverse effects.

### 3.1. Determination of Near-Field Range and the Influence Pattern of Target Azimuth on Azimuth Identification Error

#### 3.1.1. Determination of Near-Field Range

**Figure 3:**
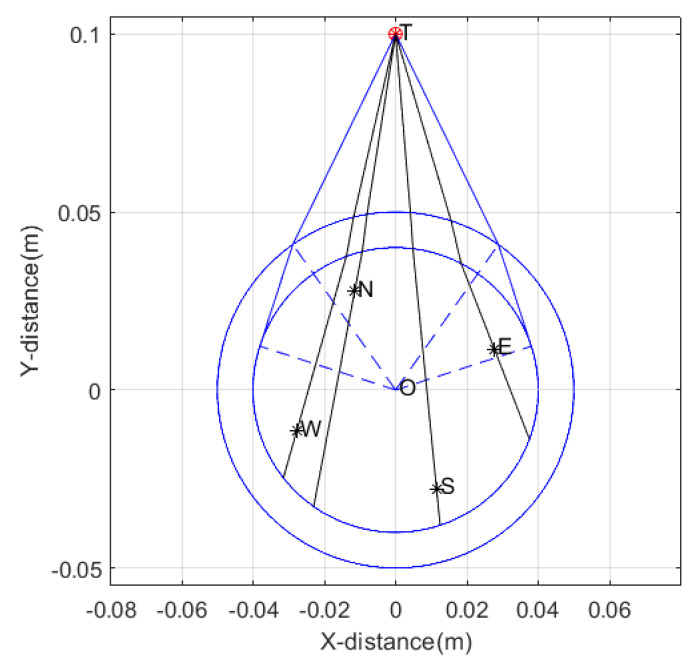
Schematic diagram of ray distribution in the near−field blind zone.

The surrounding rock is selected as granite, with a relative permittivity of 5. The fluid inside the well is oil-based mud, with a relative permittivity of 6. The probe tube is made of PVC, with a relative permittivity of 4. The four receiving antennas are point antennas. The target is located 0.1 m away from the center of the wellbore, with a wellbore radius of 5 cm, a probe tube radius of 4 cm, and an array radius of the receiving antennas of 3 cm. The azimuth angle is calculated by taking the true north direction as 0°, with the clockwise direction as the positive direction. We establish a right-handed Cartesian coordinate system with the line connecting the center of the borehole and the target as the *y*-axis, as shown in Figure 3.

The asterisk marked by T in the figure represents the target, and the signal reflected by the target is represented by the black ray. The asterisks marked by E, W, N, and S represent the east receiving antenna, west receiving antenna, north receiving antenna, and south receiving antenna, respectively. The outer blue large circle represents the borehole, and the inner blue small circle represents the sonde. The target azimuth in the schematic diagram is 22.5°.

**Figure 4:**
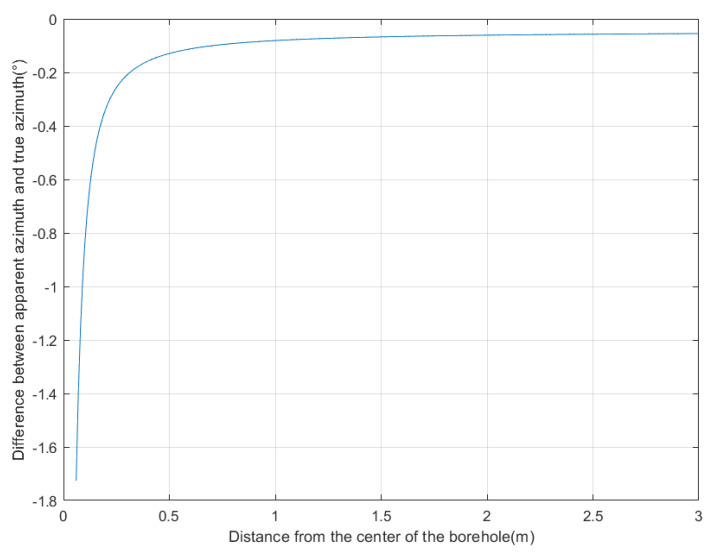
The relationship between distance and azimuth identification error.

To determine the range of the near field, we positioned the target at an azimuth angle of 22.5°. The distance between the target and the borehole center was set between 0.06 m and 3 m. We refer to the true azimuth angle of the target as the true azimuth. We determined the time delay by calculating the sequence in which signals are received by the four receiving antennas through ray tracing. Specifically, we calculated the time delay for the east–west receiving antennas based on the sequence in which they receive signals, and the time delay for the north–south receiving antennas based on the sequence in which they receive signals. Then, using the two time delays, we calculated the azimuth angle using the MUSIC algorithm, assuming a uniform medium in the far field. The calculated azimuth angle is referred to as the apparent azimuth. Using this method, we obtained the relationship between distance and azimuth identification error, as shown in Figure 4. The horizontal axis represents the distance of the target from the borehole center, and the vertical axis represents the difference between the apparent azimuth and the true azimuth. When the difference is negative, it indicates that the true azimuth is larger, and when it is positive, it indicates that the apparent azimuth is larger. As can be seen from the figure, the azimuth identification error of the MUSIC algorithm increases as the target gets closer to the borehole, and decreases as the target gets farther away. When the distance from the borehole exceeds 2 m, the decrease in azimuth identification error becomes significantly less pronounced. Therefore, the near-field blind zone range of directional borehole radar is 2 m. At the same time, we can also observe that when the distance between the target and the borehole is more than 2 m, there is still a certain degree of error. This error is caused by the refraction of electromagnetic waves due to the different dielectric constants of the borehole, probe tube, fluid inside the borehole, and surrounding rock.

#### 3.1.2. The Influence Pattern of Target Azimuth on Azimuth Identification Error

**Figure 5:**
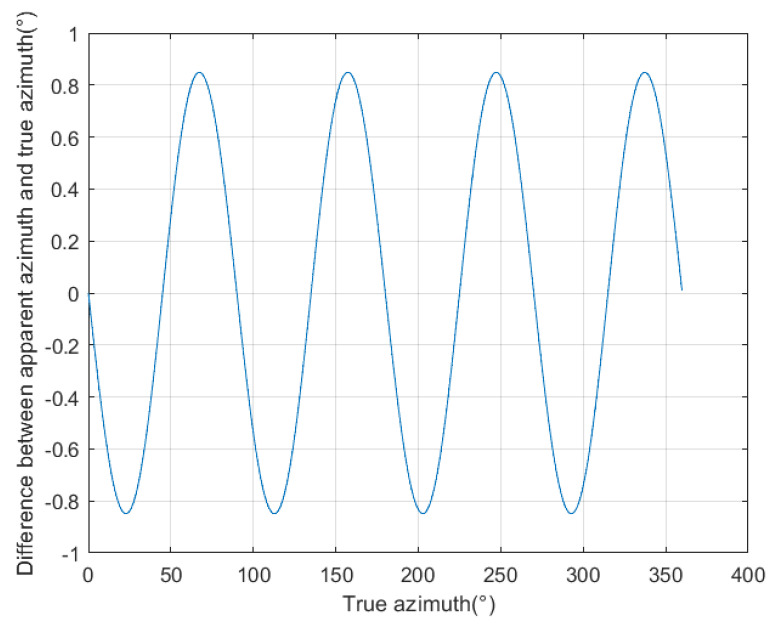
The relationship between the target’s location and the error in location identification.

We selected a target distance of 0.1 m from the borehole center, with a true azimuth angle ranging from 0° to 360° at an interval of 0.1°, to calculate the time delay of signals received by four receiving antennas. Then, using the MUSIC algorithm, we calculated the apparent azimuth angle based on the calculated time delay. Finally, we calculated the difference between the apparent azimuth angle and the true azimuth angle, and the results are shown in Figure 5. As can be seen from Figure 5, when the target azimuth angle is at 45° or a multiple of 45°, the azimuth identification error is zero. This is because these positions are symmetrical, which has minimal impact on the time delay. When the target azimuth angle is at 22.5° or an odd multiple of 22.5°, the azimuth identification error is the largest. This is because these positions are located between two symmetrical positions, resulting in the largest azimuth identification error.

### 3.2. The Influence Pattern of Antenna Center Frequency on the Received Signal of Directional Borehole Radar

**Figure 6:**
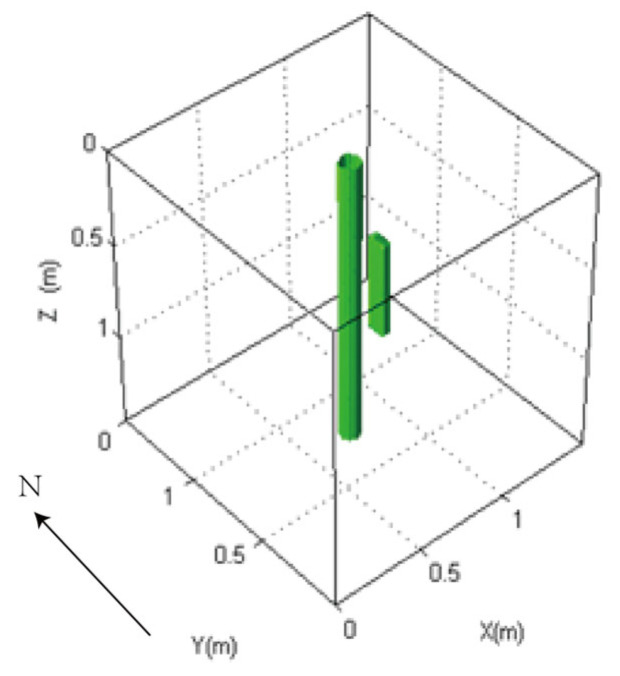
Model diagram of the impact of different antenna center frequencies on the received signal of directional borehole radar.

We employ the FDTD algorithm to numerically simulate and calculate the influence of the antenna’s center frequency on the received signal of a directional borehole radar when the target is located in the near-field blind zone. The grid size is set to 0.01 m × 0.01 m × 0.01 m, and the model dimensions are 1.5 m × 1.5 m × 1.5 m. The time window is set to 40 ns. The fluid inside the well is oil-based mud, with a relative permittivity of 6. The probe tube is made of PVC, with a relative permittivity of 4 and an electrical conductivity of 0.0001. The surrounding rock is granite, with a relative permittivity of 5 and an electrical conductivity of 0.0007. The target is a numerical fracture filled with water, with a relative permittivity of 80 and an electrical conductivity of 0.7. The target is located at a distance of 0.15 m from the borehole center. The target fracture dimensions are 0.05 m × 0.1 m × 0.5 m. Both the transmitting and receiving antennas of the directional borehole radar system are point antennas. The antenna center frequency ranges from 100 MHz to 1600 MHz. The specific model diagram is shown in Figure 6. The positive direction of y is toward the north, and the fracture is located directly to the east of the borehole. The following image uses the longitudinal component of the received signal electric field strength, namely Ez.

**Figure 7:**
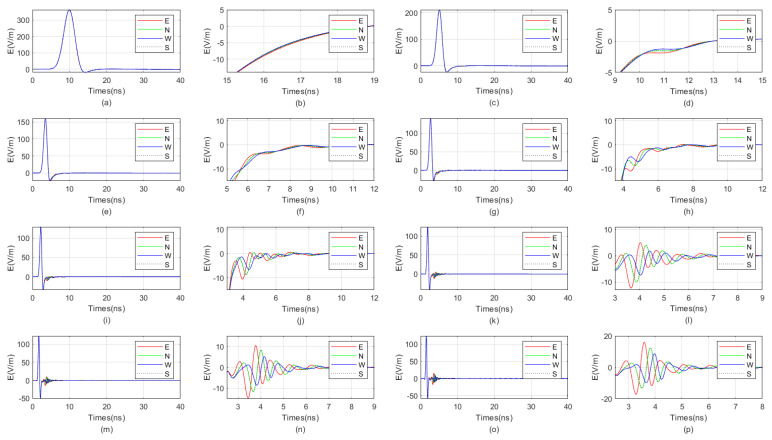
The impact of the antenna center frequency, ranging from 100 MHz to 800 MHz, on the received signal of a directional borehole radar. (**a**) Received signal at an antenna center frequency of 100 MHz; (**b**) local amplification of the received signal at an antenna center frequency of 100 MHz; (**c**) received signal at an antenna center frequency of 200 MHz; (**d**) local amplification of the received signal at an antenna center frequency of 200 MHz; (**e**) received signal at an antenna center frequency of 300 MHz; (**f**) local amplification of the received signal at an antenna center frequency of 300 MHz; (**g**) received signal at an antenna center frequency of 400 MHz; (**h**) local amplification of the received signal at an antenna center frequency of 400 MHz; (**i**) received signal at an antenna center frequency of 500 MHz; (**j**) local amplification of the received signal at an antenna center frequency of 500 MHz; (**k**) received signal at an antenna center frequency of 600 MHz; (**l**) local amplification of the received signal at an antenna center frequency of 600 MHz; (**m**) received signal at an antenna center frequency of 700 MHz; (**n**) local amplification of the received signal at an antenna center frequency of 700 MHz; (**o**) received signal at an antenna center frequency of 800 MHz; (**p**) local amplification of the received signal at an antenna center frequency of 800 MHz.

**Figure 8:**
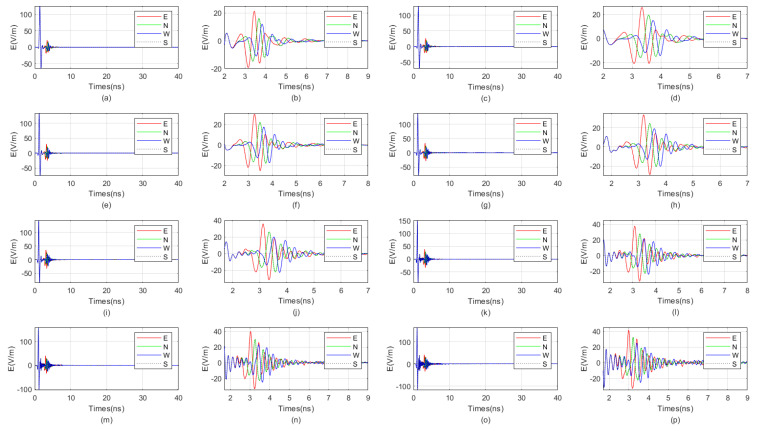
The impact of the antenna center frequency, ranging from 900 MHz to 1600 MHz, on the received signal of a directional borehole radar. (**a**) Received signal at an antenna center frequency of 900 MHz; (**b**) local amplification of the received signal at an antenna center frequency of 900 MHz; (**c**) received signal at an antenna center frequency of 1000 MHz; (**d**) local amplification of the received signal at an antenna center frequency of 1000 MHz; (**e**) received signal at an antenna center frequency of 1100 MHz; (**f**) local amplification of the received signal at an antenna center frequency of 1100 MHz; (**g**) received signal at an antenna center frequency of 1200 MHz; (**h**) local amplification of the received signal at an antenna center frequency of 1200 MHz; (**i**) received signal at an antenna center frequency of 1300 MHz; (**j**) local amplification of the received signal at an antenna center frequency of 1300 MHz; (**k**) received signal at an antenna center frequency of 1400 MHz; (**l**) local amplification of the received signal at an antenna center frequency of 1400 MHz; (**m**) received signal at an antenna center frequency of 1500 MHz; (**n**) local amplification of the received signal at an antenna center frequency of 1500 MHz; (**o**) received signal at an antenna center frequency of 1600 MHz; (**p**) local amplification of the received signal at an antenna center frequency of 1600 MHz.

The waveform diagrams of signals received by four receiving antennas with antenna center frequencies ranging from 100 MHz to 1600 MHz are shown in Figure 7 and Figure 8. It is evident from the diagrams that the lower the antenna center frequency, the wider the width of the sub-wave, but the stronger the ambiguity of the reflected signal. For example, when the antenna center frequency is below 500 MHz, it is almost impossible to determine the location of the reflected wave. The reflected wave is not only mixed with the direct wave, making it difficult to distinguish the sequence of the four received signals, but also very weak. When the antenna center frequency is at 600 MHz or 700 MHz, the sub-waves of the reflected wave can be basically distinguished, but the distortion of the sub-waves is more obvious. When the antenna center frequency is between 800 MHz and 1200 MHz, the distinction between the received signals is very good. Since the target is in the east direction, the east-facing receiving antenna receives the waveform first, while the west-facing receiving antenna receives the waveform last. The north–south-facing receiving antennas receive the waveform simultaneously and at a midpoint. When the antenna center frequency exceeds 1200 MHz, the received signals produce significant resonance. This is because, for targets within the near-field blind zone, the distance between the target and the borehole is very close. When the antenna center frequency is too high, multiple waves will be reflected back and forth between the target and the borehole, causing resonance and interfering with azimuth identification.

Therefore, based on the numerical simulation above, it can be seen that the near-field blind zone of directional drilling radar is not as it used to be, where the higher the antenna center frequency, the higher the resolution. Due to the particularity of the near-field blind zone range, which is close to the wellbore, multiple waves can become very severe when the antenna center frequency is too high, resulting in poor performance. In summary, the blind zone detection effect is best when the antenna center frequency is between 800 MHz and 1200 MHz.

**Figure 9:**
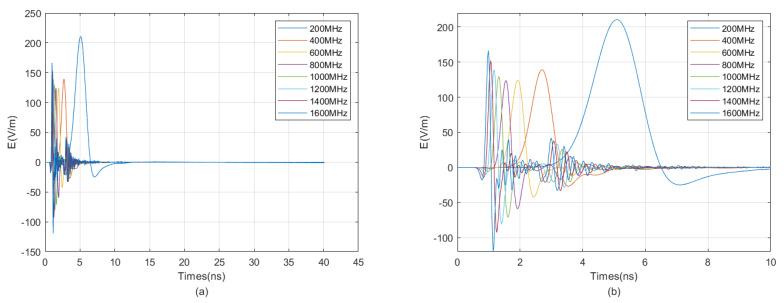
Signal reception diagram of east-facing receiving antennas at various frequencies. (**a**) Global map; (**b**) local enlarged view.

We selected east-facing receiving antennas to receive signals for aggregation and comparison, as shown in Figure 9. From the figure, it can be observed that as the antenna frequency increases, the sub-wave becomes narrower, and the differentiation of reflected signals gradually improves, while the intensity of direct wave signals gradually decreases. When the antenna frequency is too high, the intensity of direct wave signals rises again, and multiple waves become evident. Therefore, the optimal antenna center frequency ranges from 800 MHz to 1200 MHz.

### 3.3. The Influence Pattern of the Relative Permittivity of the Fluid in the Borehole on the Received Signal

**Figure 10:**
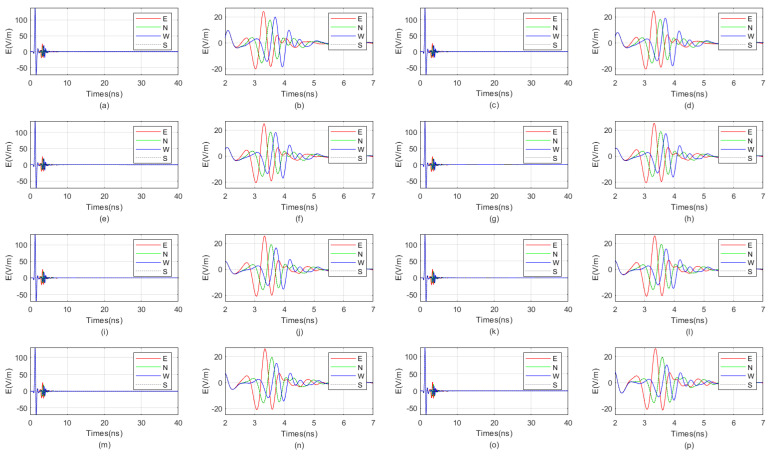
When the target is located in the blind zone, the influence of the relative permittivity of the fluid in the borehole, ranging from 3 to 7, on the received signal of the directional borehole radar. (**a**) The received signal has a relative permittivity of 3 for the fluid inside the borehole; (**b**) local amplification diagram of the received signal with a relative permittivity of 3 for the fluid in the borehole; (**c**) the received signal has a relative permittivity of 3.5 for the fluid inside the borehole; (**d**) local amplification diagram of the received signal with a relative permittivity of 3.5 for the fluid in the borehole; (**e**) the received signal has a relative permittivity of 4 for the fluid inside the borehole; (**f**) local amplification diagram of the received signal with a relative permittivity of 4 for the fluid in the borehole; (**g**) the received signal has a relative permittivity of 4.5 for the fluid inside the borehole; (**h**) local amplification diagram of the received signal with a relative permittivity of 4.5 for the fluid in the borehole; (**i**) the received signal has a relative permittivity of 5 for the fluid inside the borehole; (**j**) local amplification diagram of the received signal with a relative permittivity of 5 for the fluid in the borehole; (**k**) the received signal has a relative permittivity of 5.5 for the fluid inside the borehole; (**l**) local amplification diagram of the received signal with a relative permittivity of 5.5 for the fluid in the borehole; (**m**) the received signal has a relative permittivity of 6 for the fluid inside the borehole; (**n**) local amplification diagram of the received signal with a relative permittivity of 6 for the fluid in the borehole; (**o**) the received signal has a relative permittivity of 7 for the fluid inside the borehole; (**p**) local amplification diagram of the received signal with a relative permittivity of 7 for the fluid in the borehole.

**Figure 11:**
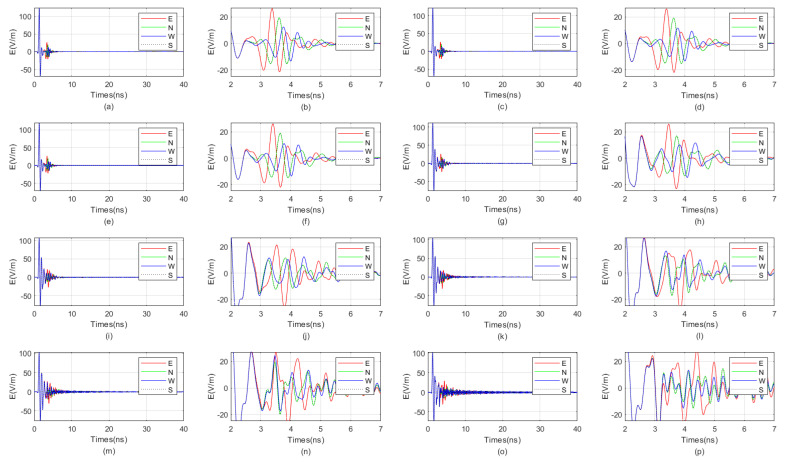
When the target is located in the blind zones, the influence of the relative permittivity of the fluid in the borehole, ranging from 8 to 40, on the received signal of the directional borehole radar. (**a**) The received signal has a relative permittivity of 8 for the fluid inside the borehole; (**b**) local amplification diagram of the received signal with a relative permittivity of 8 for the fluid in the borehole; (**c**) the received signal has a relative permittivity of 9 for the fluid inside the borehole; (**d**) local amplification diagram of the received signal with a relative permittivity of 9 for the fluid in the borehole; (**e**) the received signal has a relative permittivity of 10 for the fluid inside the borehole; (**f**) local amplification diagram of the received signal with a relative permittivity of 10 for the fluid in the borehole; (**g**) the received signal has a relative permittivity of 15 for the fluid inside the borehole; (**h**) local amplification diagram of the received signal with a relative permittivity of 15 for the fluid in the borehole; (**i**) the received signal has a relative permittivity of 20 for the fluid inside the borehole; (**j**) local amplification diagram of the received signal with a relative permittivity of 20 for the fluid in the borehole; (**k**) the received signal has a relative permittivity of 25 for the fluid inside the borehole; (**l**) local amplification diagram of the received signal with a relative permittivity of 25 for the fluid in the borehole; (**m**) the received signal has a relative permittivity of 30 for the fluid inside the borehole; (**n**) local amplification diagram of the received signal with a relative permittivity of 30 for the fluid in the borehole; (**o**) the received signal has a relative permittivity of 40 for the fluid inside the borehole; (**p**) local amplification diagram of the received signal with a relative permittivity of 40 for the fluid in the borehole.

We investigate the influence of the relative permittivity of the fluid inside the borehole on the received signal of the directional drilling radar. The numerical simulation parameters are the same as those in Section 3.2. The relative permittivity of the fluid inside the borehole varies from 3 to 40. The received signal diagrams are shown in Figure 10 and Figure 11. It is evident from the figures that when the relative permittivity of the fluid inside the borehole is below 10, the waveform distinction of the reflected wave is very good. When the relative permittivity of the fluid inside the borehole is above 15, the received signal gradually begins to exhibit increasingly strong resonance.

**Figure 12:**
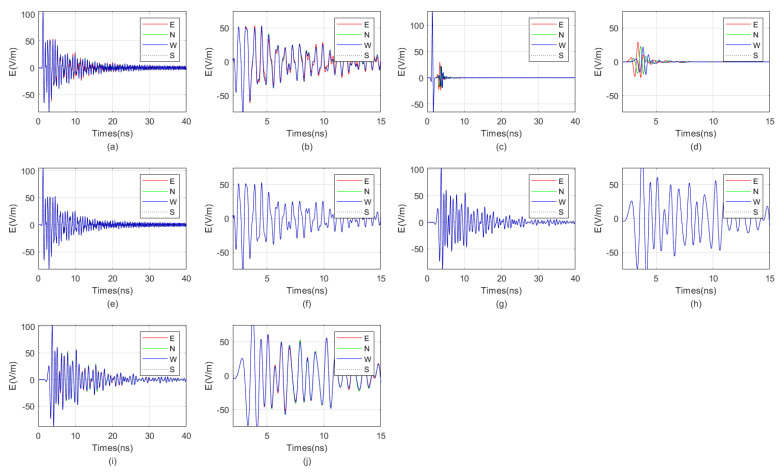
Numerical simulation results of the cause of resonance when the relative permittivity of the fluid in the borehole is 80. There are (**a**) “borehole, probe tube, and target” conditions; (**b**) local magnification of “borehole, probe tube, and target” conditions; (**c**) “no borehole, no probe tube, but with target” conditions; (**d**) local magnification of “no borehole, no probe tube, but with target” conditions; (**e**) “with a wellbore and a probe tube, but no target” conditions; (**f**) local magnification of “with a wellbore and a probe tube, but no target” conditions; (**g**) “with a borehole, no probe tube, and no target” conditions; (**h**) local magnification of “with a borehole, no probe tube, and no target” conditions; (**i**) “with a borehole but no probe tube, there is a target” conditions; (**j**) local magnification of “with a borehole but no probe tube, there is a target” conditions.

To further investigate the causes of resonance, numerical simulations were conducted for the case where the relative permittivity of the fluid inside the borehole is 80. Simulations were carried out for three scenarios: no borehole, no probe tube, and with a target; with a borehole and a sonde, but without a target; and with a borehole, no sonde, and no target. The simulation results are shown in Figure 12. It can be observed from the figure that resonance occurs when the relative permittivity of the fluid inside the borehole is 80. However, in the scenario without a borehole, sonde, and with a target, resonance disappears, indicating that the occurrence of resonance is related to the borehole. In the scenario with a borehole, sonde, and without a target, resonance still exists, suggesting that the occurrence of resonance is not related to the target. Therefore, resonance can only originate from within the borehole. In the scenario with a borehole, no sonde, and no target, resonance still persists, indicating that resonance originates from multiple reflections between the borehole walls.

### 3.4. The Influence Pattern of Target Distance on the Received Signal of Directional Borehole Radar

**Figure 13:**
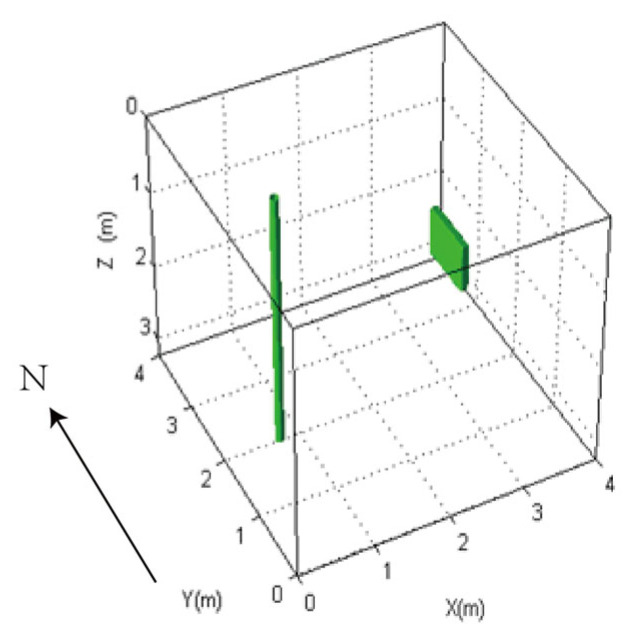
Model diagram of the influence of target distance on the received signal of directional borehole radar.

**Figure 14:**
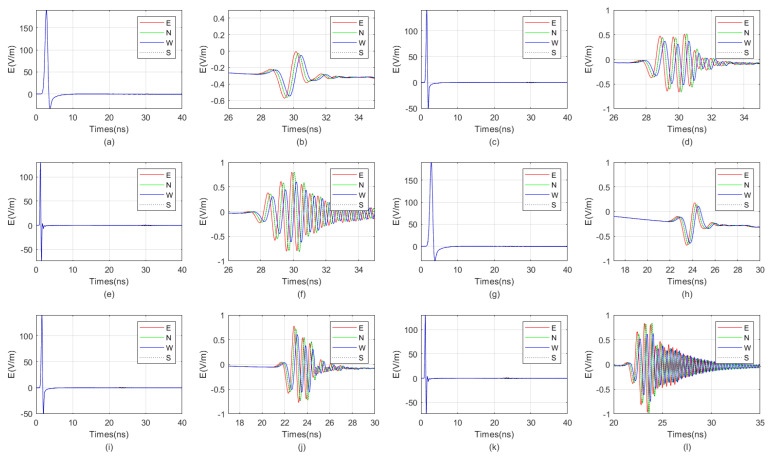
When the target distances are 2.25 m and 1.75 m, the directional borehole radar receives signals. (**a**) When the target distance is 2.25 m, the antenna receives a signal with a center frequency of 300 MHz. (**b**) When the target distance is 2.25 m, the antenna center frequency is 300 MHz, and the received signal is locally amplified. (**c**) When the target distance is 2.25 m, the antenna receives a signal with a center frequency of 600 MHz. (**d**) When the target distance is 2.25 m, the antenna center frequency is 600 MHz, and the received signal is locally amplified. (**e**) When the target distance is 2.25 m, the antenna receives a signal with a center frequency of 900 MHz. (**f**) When the target distance is 2.25 m, the antenna center frequency is 900 MHz, and the received signal is locally amplified. (**g**) When the target distance is 1.75 m, the antenna receives a signal with a center frequency of 300 MHz. (**h**) When the target distance is 1.75 m, the antenna center frequency is 300 MHz, and the received signal is locally amplified. (**i**) When the target distance is 1.75 m, the antenna receives a signal with a center frequency of 600 MHz. (**j**) When the target distance is 1.75 m, the antenna center frequency is 600 MHz, and the received signal is locally amplified. (**k**) When the target distance is 1.75 m, the antenna receives a signal with a center frequency of 900 MHz. (**l**) When the target distance is 1.75 m, the antenna center frequency is 900 MHz, and the received signal is locally amplified.

**Figure 15:**
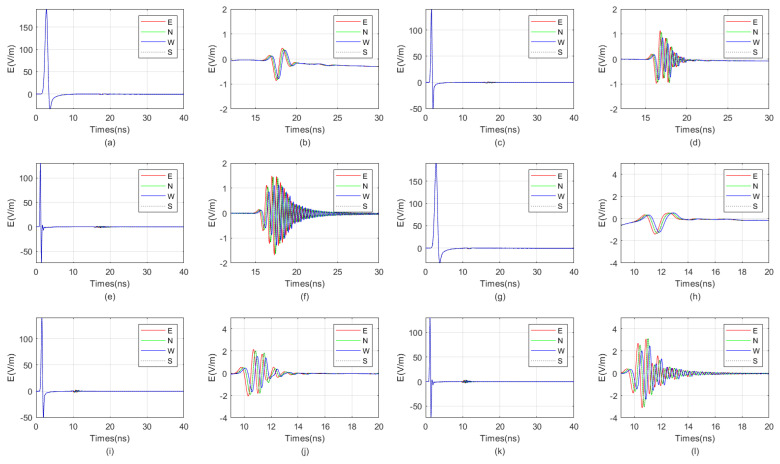
When the target distances are 1.25 m and 0.75 m, the directional borehole radar receives signals. (**a**) When the target distance is 1.25 m, the antenna receives a signal with a center frequency of 300 MHz. (**b**) When the target distance is 1.25 m, the antenna center frequency is 300 MHz, and the received signal is locally amplified. (**c**) When the target distance is 1.25 m, the antenna receives a signal with a center frequency of 600 MHz. (**d**) When the target distance is 1.25 m, the antenna center frequency is 600 MHz, and the received signal is locally amplified. (**e**) When the target distance is 1.25 m, the antenna receives a signal with a center frequency of 900 MHz. (**f**) When the target distance is 1.25 m, the antenna center frequency is 900 MHz, and the received signal is locally amplified. (**g**) When the target distance is 0.75 m, the antenna receives a signal with a center frequency of 300 MHz. (**h**) When the target distance is 0.75 m, the antenna center frequency is 300 MHz, and the received signal is locally amplified. (**i**) When the target distance is 0.75 m, the antenna receives a signal with a center frequency of 600 MHz. (**j**) When the target distance is 0.75 m, the antenna center frequency is 600 MHz, and the received signal is locally amplified. (**k**) When the target distance is 0.75 m, the antenna receives a signal with a center frequency of 900 MHz. (**l**) When the target distance is 0.75 m, the antenna center frequency is 900 MHz, and the received signal is locally amplified.

**Figure 16:**
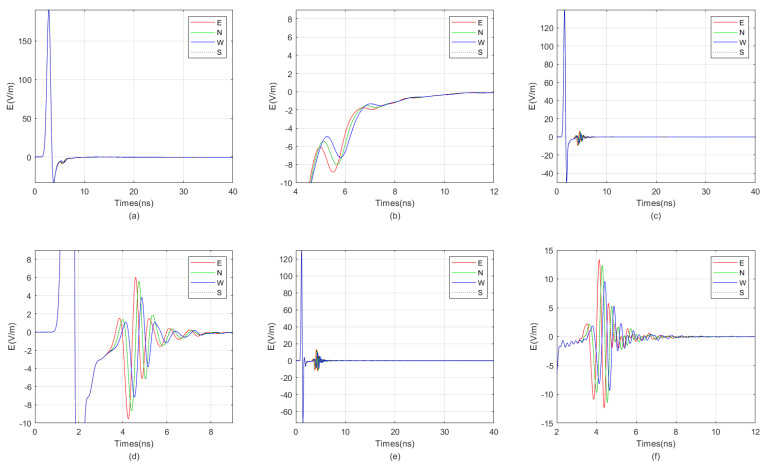
When the target distances are 0.25 m, the directional borehole radar receives signals. (**a**) When the target distance is 0.25 m, the antenna receives a signal with a center frequency of 300 MHz. (**b**) When the target distance is 0.25 m, the antenna center frequency is 300 MHz, and the received signal is locally amplified. (**c**) When the target distance is 0.25 m, the antenna receives a signal with a center frequency of 600 MHz. (**d**) When the target distance is 0.25 m, the antenna center frequency is 600 MHz, and the received signal is locally amplified. (**e**) When the target distance is 0.25 m, the antenna receives a signal with a center frequency of 900 MHz. (**f**) When the target distance is 0.25 m, the antenna center frequency is 900 MHz, and the received signal is locally amplified.

We employed the FDTD algorithm to investigate the impact of target distance on the directional borehole radar’s receiving antenna. The grid size was 0.0125 m × 0.0125 m × 0.0125 m, and the model size was 4 m × 4 m × 3.25 m. The positive direction of the *Y*-axis was defined as true north. The target was located directly east of the borehole. The borehole radius was 5 cm, the probe tube radius was 3.75 cm, and the array radius of the receiving antenna was 2.5 cm. The relative permittivity of the fluid inside the borehole was 6. The surrounding rock was granite, and the target was located in the east with water as the filler. The electrical parameters of granite and water were as previously described. The target dimensions were 0.1 m × 0.8 m × 0.6 m. The model is shown in Figure 13. We simulated the impact of varying target distances from the borehole on the received signals at antenna center frequencies of 300 MHz, 600 MHz, and 900 MHz, respectively. The results are shown in Figure 14, Figure 15, and Figure 16.

From the result graph, it can be observed that the greater the distance, the weaker the waveform distortion of the received signal due to the unevenness around the borehole. Especially in the far-field region, the borehole, sonde, and fluid inside the borehole exert almost no distortion on the received signal. Simultaneously, the higher the frequency, the more pronounced the multiple waves generated for targets such as water-bearing fractures. In other words, for water-bearing fractures, higher frequency does not necessarily equate to higher resolution. When the frequency is excessively high, it can generate a large number of multiple waves, leading to resonance. This results in a wider width of the reflected wave, which in turn reduces resolution. The graph also indicates that lower frequencies correspond to lower signal strength of the reflected wave. Additionally, the closer the target is, the less impact multiple waves caused by excessively high antenna frequency have. Therefore, the optimal center frequency of the antenna varies with different target distances. Generally, the farther the target is from the borehole, the lower the optimal center frequency of the antenna. In the example provided, when the distance from the borehole is 2.25 m, the optimal center frequency of the antenna is 300 MHz, while when the distance is 0.25 m, the optimal center frequency is 900 MHz.

## 4. Discussion

This article conducts a numerical simulation of the impact of blind zones on directional borehole radar using the FDTD method and obtains the following rules.

As the distance between the target and the borehole increases, the azimuth identification error continuously decreases. This is because the azimuth identification algorithm of the directional drilling radar is based on the far-field assumption. The farther the target is from the borehole, the more it satisfies the premise of the azimuth identification algorithm, naturally resulting in a smaller error. At the same time, when the target is located at azimuth angles of 45° and multiples of 45°, the azimuth identification error is zero. This is because these positions are symmetrical, which minimizes the impact on time delay. In this case, the time delay between the east–west receiving antenna and the north–south receiving antenna is either zero or equal, resulting in great accuracy. The azimuth identification algorithm relies on time delay, so the error is almost zero under these conditions. When the target azimuth angle is 22.5° or an odd multiple of 22.5°, the azimuth identification error is the largest. This is because these positions are between two symmetrical positions, with relatively large refraction angles, causing the most severe distortion to the ray path, resulting in the largest azimuth identification error.

The center frequency of the antenna also has a significant impact on the received signal. When the center frequency of the antenna is too low, the reflected signal is very insignificant due to the wide sub-wave. At the same time, when the center frequency of the antenna is too high, it is also not suitable for situations where the target is in the near-field blind zone. This is because when the center frequency of the antenna is too high, it causes electromagnetic waves to reflect back and forth between the target and the borehole, resulting in multiple wave resonances. This severely interferes with the determination of the target distance. Therefore, there is an optimal range for the center frequency of the antenna in the near-field blind zone, which is between 800 MHz and 1200 MHz.

The relative permittivity of the fluid inside the borehole is also an important factor affecting the received signal of the directional borehole radar. When the relative permittivity of the fluid inside the borehole is below 10, the distinguishability of the received signal is very good. When the relative permittivity of the fluid inside the borehole exceeds 15, resonance occurs in the received signal, and the higher the permittivity, the more pronounced the resonance. Numerical simulation verifies that the occurrence of resonance is caused by multiple propagations of electromagnetic waves between the well walls. Therefore, in reality, mud with a relative permittivity below 10 is oil-based mud, so choosing oil-based mud is the best choice.

The optimal antenna center frequency varies with the distance from the borehole. When the antenna center frequency is high, multiple waves are easily generated inside the target, seriously affecting the determination of the target’s position. However, when the target is closer to the borehole, these multiple waves become less pronounced. Numerical simulations have verified that the optimal antenna center frequency is 900 MHz when the target is 2.25 m away from the well and 300 MHz when the target is 0.25 m away. The results of this numerical simulation can be summarized in Table 1.

Due to the fact that both the transmitting and receiving antennas in this numerical simulation experiment are point antennas, but in reality, the shape of the antenna has a significant impact on the receiving signal of directional drilling radar, the shape of the transmitting and receiving antennas can be taken into account in the next numerical simulation experiment.

## 5. Conclusions

After calculation and numerical simulation verification, it is found that within a range of 2 m from the well, the azimuth identification error is relatively large, which can be defined as the near-field region. The direction of true north is taken as 0°, and the clockwise direction is considered as the positive direction. When the target azimuth angle is 45° or a multiple of 45°, the azimuth identification error is zero. When the target azimuth angle is 22.5° or a multiple of 22.5°, the azimuth identification error is at its maximum. In the near-field blind zone, the optimal frequency range for the antenna is between 600 MHz and 1200 MHz. When the relative permittivity of the fluid inside the borehole is higher than 15, the received signal will exhibit significant resonance. Therefore, using oil-based mud is significantly better than water-based mud. The most suitable antenna center frequency varies with the distance from the borehole. When the antenna center frequency is higher, it is prone to generating multiple waves inside the target, seriously affecting the determination of the target’s position. Numerical simulation verification shows that when the target is at a distance of 2.25 m from the borehole, the optimal antenna center frequency is 900 MHz, and when the target is at a distance of 0.25 m from the borehole, the optimal antenna center frequency is 300 MHz.

## Figures and Tables

**Table 1:** Conclusion of this numerical simulation.

Blind Zones Range	Maximum Error Direction	Best Antenna Center Frequency	Optimal Borehole Fluid	The Farther the Target Distance
2 m (1 GHz)	Odd multiples of 22.5°	600 MHz–1100 MHz	Oil-base mud	the lower the optimal frequency

## Data Availability

Data are contained within the article.
